# Effect of Roasting on the Antioxidant Activity, Phenolic Composition, and Nutritional Quality of Pumpkin (*Cucurbita pepo* L.) Seeds

**DOI:** 10.3389/fnut.2021.647354

**Published:** 2021-03-10

**Authors:** Mengyao Peng, Dan Lu, Jie Liu, Bo Jiang, Jingjing Chen

**Affiliations:** ^1^State Key Laboratory of Food Science and Technology, Jiangnan University, Wuxi, China; ^2^China-Canada Joint Lab of Food Nutrition and Health (Beijing), Beijing Technology and Business University (BTBU), Beijing, China

**Keywords:** pumpkin seeds, roasting, antioxidant activity, phenolic acids, volatile matter

## Abstract

In recent years, with the increasing awareness of health concerns and environment protection needs, there is a growing interest for consumers to choose plant-based food diets compared with those made from animal origin. Pumpkin seed is an excellent dietary source for protein, oil, and some essential micronutrients. Raw pumpkin seed may have a compromised flavor, color, as well as digestibility. Therefore, the objective of present study is to study the influence of roasting (120, 160, and 200°C for 10 min) on the phenolics content, flavonoids content, antioxidant property, fatty acids, and volatile matter composition, as well as protein profile of pumpkin seeds. Our results indicated that, total phenolic compounds, total flavonoids content, as a consequence, total antioxidant capacity increased as the roasting temperature increased. Maillard reaction products and lipid peroxidation products were identified, especially from those pumpkin seeds roasted at high temperature. In the meantime, the composition and content of fatty acids did not change significantly after roasting. The results of electrophoresis and particle size analysis showed that the optimum roasting temperature was 160°C to obtain protein with better nutritional quality. The findings of this study may contribute to the utilization of pumpkin seed component in plant-based diets with increased nutritional quality.

## Introduction

Pumpkin belongs to the family Cucurbitaceae, is commonly classified into five species: *C. pepo, C. moschata, C. mixta, C. maxima*, and *C. stilbo* (1). In recent years, pumpkin has been widely applied to healthcare industry because of its rich nutraceutical and therapeutic value. Although pumpkin seeds are accepted as snacks or added as protein supplement in some regions, they are generally wasted after extracting oil (2). As pumpkin by-products, the seeds receive growing attention as functional food owing to their biological and pharmacological potentials. In addition to carbohydrate, protein and other common nutrients, vitamin, carotenoids, squalene, phytosterols, cucurbitacin, and phenolic compounds can be also found in pumpkin seeds (3). Pumpkin extracts have the potential to prevent prostate cancer and urinary disorders (4), effective for ameliorating hypertension and diabetes (5), showing anthelmintic (6), anti-hypercholesterolemia, and antitumor (7) activities.

Phenol, with one or more aromatic rings and hydroxyl groups, are widely found in plant tissues and have the functions of antioxidation, pathogen resistance, plant color formation, and so on (8). Although plant polyphenols have been extensively studied as natural antioxidants, there are few literatures on phenolic compounds in pumpkin seeds. The phenolic acids in pumpkin (*Cucurbita pepo*) seeds and hulls have been investigated and it indicated that p-hydroxybenzoic acid was the dominant phenolic acids in the hull-less seeds with green skin. Besides, there are also caffeic, ferulic, and vanillic acids (9). Fourteen flavonoids and six phenolic acids were identified in raw *Cucurbita moschata* D. seeds by Enneb et al. (10). P-hydroxybenzoic acid, caffeic acid, ferulic acid and vanillic acid were proved to be the main phenolic acids in pumpkin seeds as reported by Dotto and Chacha (1).

Roasting is a traditional processing method, which can enhance nutritional value and sensory quality of various foods. Furthermore, roasting affects the antioxidant capacity of products. Roasting is crucial to generate the aroma components of pumpkin seed oil and roasting temperatures required to be higher than 100°C (11). The composition and concentration of volatile compounds are significantly influenced by roasting temperature. At lower temperature, the content of aldehydes and alcohols tend to be higher. High temperature cuaused the formation of different pyrazines, which were considered to be the key aroma components of pumpkin seed oil (11–13). A study with headspace solid phase microextraction followed by gas chromatography and mass spectrometry (HS-SPME-GS-MS) measured the volatile components of roasted pumpkin seeds directly instead of pumpkin seed oil. They found that polycyclic aromatic hydrocarbon (PAHs), which may be genotoxic and carcinogenesis, was generated at 150°C (13).

Plant protein has been studied as a substitute for animal protein because of their economic efficiency in recent years (14). The protein content of pumpkin seed cake (after oil extraction) was high and the amino acid composition was similar to that of soybean protein (15). Many studies have shown that pumpkin seed protein contains all essential amino acids, and lysine is the first limiting amino acid, with arginine and glutamic acid as the main components (16–18). Consequently, pumpkin seeds are considered to be a good source of plant protein. Studies on roasting and nutritional properties of pumpkin seed are relatively scarce. Investigation on the effect of roasting on pumpkin seed protein profile may promote its application in food systems and improve its commercial value.

The objective of present study is to investigate the influence of roasting (120, 160, and 200°C for 10 min) on the phenolics content, flavonoids content, antioxidant property, fatty acids, and volatile matter composition, as well as protein profile of pumpkin seeds. The findings of this study may contribute to understand the relationship between roasting and nutritional quality of pumpkin seed, as well as the utilization of pumpkin seed component in plant-based diets.

## Materials and Methods

### Materials

Pumpkin seeds (*Cucurbita pepo L*.) were provided by Haichuansanxin Food Company (Beitun, China). They were kept in vacuum pouches at −4°C for subsequent research. The nutritional composition of the used pumpkin seeds was 34.25% proteins, 46.15% fats, 4.39% moisture, 4.52% ash, and 3.01% carbohydrates. Methanol HPLC grade, 2,2-diphenyl-1-picrylhydrazyl (DPPH) aluminum chloride, gallic acid and rutin, p-hydroxybenzoic, epicatechin, caffeic, p-coumaric, ferulic were purchased from Sinopharm Chemical Reagent Co., Ltd. (Shanghai, China). All other reagents were analytical grade and obtained from Chemical Reagent Co., Ltd. (Shanghai, China).

### Roasting of Pumpkin Seeds

The pumpkin seeds were spread uniformly in preheated trays and were roasted at 120, 160, and 200°C for 10 min, respectively, using a NB-HM3810 oven (Panasonic Corporation, Osaka, Japan). Both raw pumpkin seeds and roasted ones were grounded into flours using a YF-1000 blender (Yongli Pharmaceutical Mechanic, Hangzhou, Zhejiang, China). The flours were then passed through a 60-mesh screen and stored in a −80°C freezer for further use.

### Antioxidant Properties

#### Extraction of Phenolic Compounds

The phenolic compounds in roasted pumpkin seed flours were extracted in reference to the method of Bhinder (19). Methanol solution (80%, v/v) was used as solvent to extract free and bound phenolics (mass volume ratio 1:15) twice. The extracts were combined, filtered, and used for determining antioxidant activity and phenolic acids.

#### Phenolic Content

Folin-Ciocalteu (FC) method was used to measure the total phenol content of pumpkin seeds according to method published by Saavedra et al. (20) with some modification. Two hundred and fifty microliters of pumpkin seeds extract was diluted to 3.8 mL, 300 μL FC reagent was added and reacted for 8 min. Then 20% sodium carbonate (900 μL) was added and stound for 1 h in dark, followed by the measure of the absorbance at 750 nm wavelength using P7 Double Bean UV-Visible Spectrophotomer (Meipuda instrument Co., LTD, Shanghai, China). Under the same conditions, the standard curve of gallic acid (1, 2, 3, 4, 5, and 6 μg/mL) was drawn. Free phenols, bound phenols and total phenols were expressed in terms of the content of gallic acid (mg) per gram of pumpkin seeds (mg GAE/g).

#### Flavonoid Content

The concentrations of flavonoids in pumpkin seeds were determined by sodium nitrite-aluminum chloride complexation spectrophotometry following the procedure reported by Shen et al. (21). The absorbance was determined at 510 nm on P7 Double Bean UV-Visible Spectrophotomer (Meipuda instrument Co., Ltd., Shanghai, China) and the standard curves of rutin (16, 20, 26, 30, 36, and 40 ppm) were drawn. The rutin content per gram of pumpkin seeds (mg RE/g) was used to represent the different flavonoid content.

#### Antioxidant Activity

DPPH free radical assay was used to evaluate the antioxidant activity following by the method of Sánchez-Moreno et al. (22). The absorbance of the extract reacting with 0.1 mmol/L of DPPH methanol solution was determined at 517 nm on P7 Double Bean UV-Visible Spectrophotomer (Meipuda instrument Co., Ltd., Shanghai, China) after standing in dark for 30 min, zero the blank with methanol solution, and calculate the DPPH free radical scavenging capacity. At the above wavelength, the absorbance of Trolox standard solution was determined and the calibration curve was drawn. The antioxidant activity of pumpkin seeds was presented as μmol Trolox equivalent per gram of pumpkin seeds (μmol TE/g).

### Phenolic Acids Composition

Phenolic acids were determined with Agilent 1100 HPLC equipped with an autosampler (Agilent, California, USA), UV-visible detector at 280 nm, C18 column (5 μm, 4.6 mm × 250 mm). Mobile phases A and B were 1% acetic acid and chromatographic methanol, respectively. The gradient elution procedure was as follows: from 0 to 10 min, 80% A; from 10 to 25 min, 80–60% A; from 25 to 35 min, 50% A; from 35 to 40 min, 50–30% A; from 40 to 45 min, 90% A. Phenolic acids were identified by comparing the retention times of mixed standard chromatogram and the relative content were calculated through a normalization procedure.

### Fatty Acids Composition

The composition of fatty acids in pumpkin seeds were measured according to the method of Zhang et al. (23) using a GC 2030 AF gas chromatography equipped with automatic injector (Shimadzu Corporation, Kyoto, Japan). The temperature of the column was raised from 80 to 250°C by temperature programmed method. The temperature of flame ionization detector (FID) was also 250°C. Identification of fatty acids was completed by comparing the retention indices with NSIT Library and calculating relative content by peak area normalization.

### Composition of Volatile Matters

The composition of volatile matters was investigated using Pegasus BT gas chromatography time of flight mass spectrometer (Leco Corporation, St. Joseph, USA). The followings are the chromatographic conditions of GC-MS. The initial DB-FFAP (30 m × 0.25 mm × 0.25 μm) column temperature was 50°C, holding for 2 min, then increased to 150 at 3°C/min, holding for 1 min and increased to 250 at 5°C/min. The injection port and the ion source temperature were both 250°C. Electron impact ion was used (70 eV) to scan a mass ranging from 33 to 450 amu. According to the area normalization method, the contents of volatile compounds were expressed as relative percentages. The volatile compounds were identified by computer and matched with NSIT and Wiley Library. The relative percentage content was calculated by peak area normalization.

### Protein Extraction

Pumpkin seed protein was extracted by alkali dissolution and acid precipitation method (24). The pumpkin seed powder defatted with n-hexane was stirred in pH 11 sodium hydroxide solution for 2 h and then adjust to pH 5 with 1 mol/L HCl. The precipitate obtained after centrifugation was freeze-dried by a 6 L freeze dryer (Laboconcor Corporation, Kansas, MO, USA).

#### SDS-PAGE

The protein components of roasted pumpkin seeds were analyzed by sodium dodecyl sulfate polyacrylamide gel electrophoresis (SDS-PAGE). The concentration of electrophoresis gel was 12.5% and that of concentrated gel was 4%. The pumpkin seed protein dissolved in the pH 7.0 phosphate buffer solution whose content was 5 mg/mL determined by microplate (Molecular Devices, LLC., California, USA)was mixed with the sample buffer (5 ×, 250 mM Tris-HCl, pH 6.8, 10% SDS, 0.5% bromophenol blue, 50% glycerol, 5% β- mercaptoethanol) at the volume ratio of 4:1. The mixed sample was soaked in boiling water for 10 min and was loaded (10 μL). The gels run at the voltage of 120 V with Tris-Glycine buffer (25 mM Tris, 250 mM glycine, 0.1% SDS). After electrophoresis, the SDS-PAGE gel was stained with Coomassie brilliant blue G-250 for 2 h, and compare with a protein maker by ChemiDocXRS+ gel imaging analysis system (Bio-Rad Laboratories, Inc., California, USA) after decolorization. The protein marker purchased from Epizyme Biotechnology Co., Ltd (Shanghai, China) consists of 11 protein bands ranging from 10 to 250 kDa.

#### Particle Size

The particle size of pumpkin seed protein in pure water (1 mg/mL) was measured after 0 h and 4 h using Nano Brook Omni particle size and zeta potential analyser (Brookhaven Instruments Corporation, New York, USA) to analyse the stability of the protein from pumpkin seed roast at different temperatures.

### Statistical Analysis

The assay results were expressed as average value ± standard deviation of three replicates. Comparisons among the pumpkin seeds from each treatment were performed by ANOVA using IBM SPSS Statistics 26.0. Results with *p* < 0.05 were considered significantly different.

## Results and Discussion

### Polyphenolic Content and Antioxidant Properties

**Figure 1:**
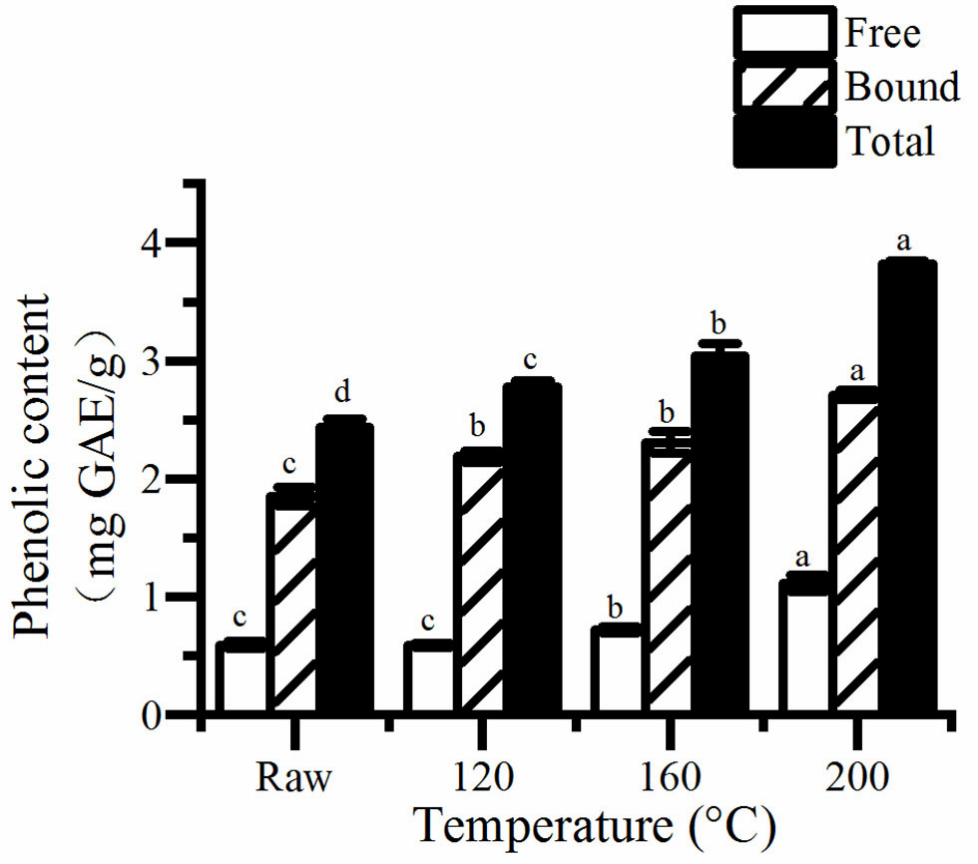
The free, bound, and total content of phenolic in roasted pumpkin seeds. GAE, gallic acid equivalents. Different small letters (a–d) indicate that there are significant differences in the phenolic content at different roasting temperatures (*P* < 0.05). Raw, raw pumpkin seed.

**Figure 2:**
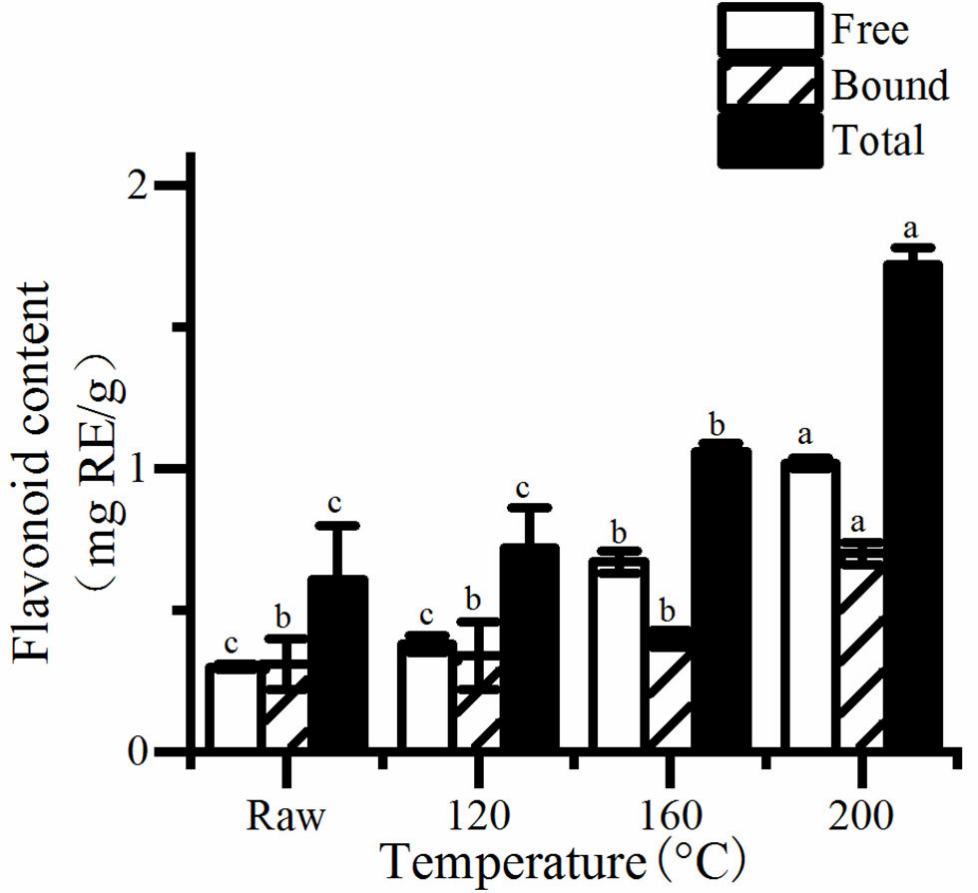
The free, bound, and total content of flavonolid in roasted pumpkin seeds. RE, rutin equivalents. Different small letters (a–d) indicate that there are significant differences in the flavonid content at different roasting temperatures (*P* < 0.05). Raw, raw pumpkin seed.

**Figure 3:**
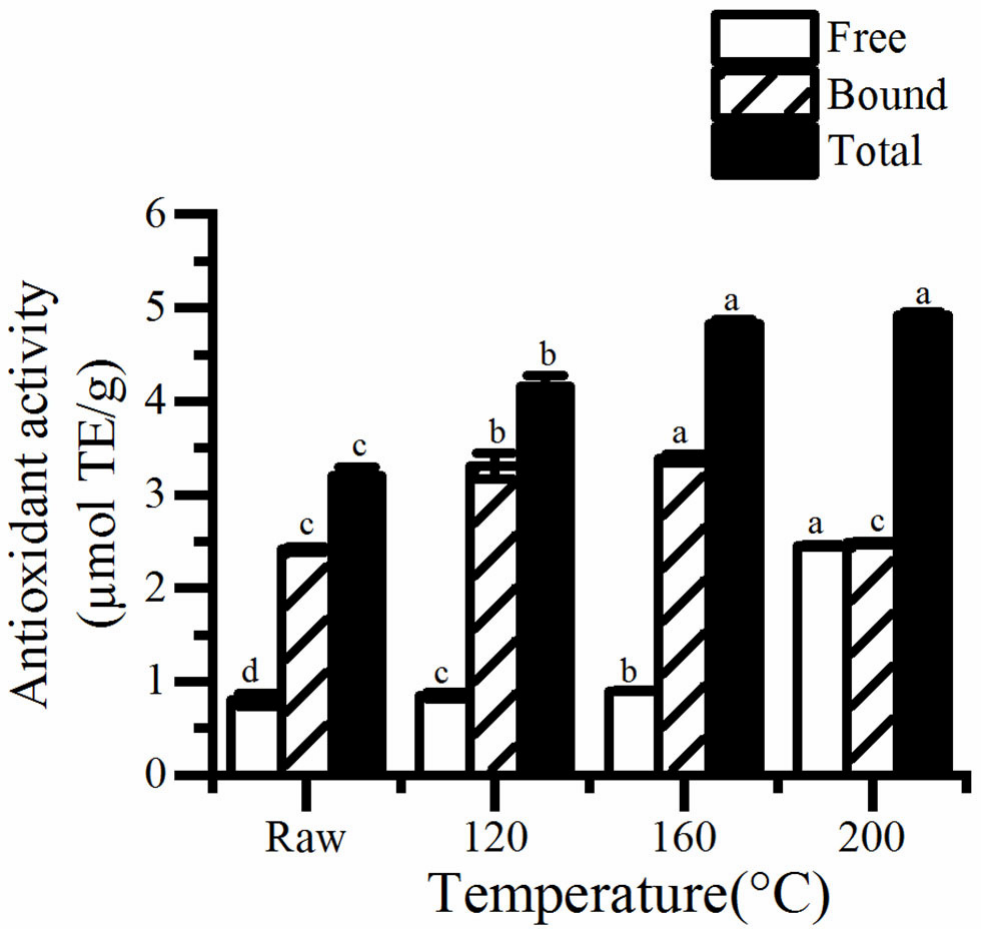
The free, bound, and total antioxidant activity in roasted pumpkin seeds. TE, Trolox equivalents. Different small letters (a–d) indicate that there are significant differences in the antioxidant activity at different roasting temperatures (*P* < 0.05). Raw, raw pumpkin seed.

The concentrations of free, bound and total phenols and flavonoids, as well as antioxidant activity are shown in Figures 1–3. The result showed that the concentration of bound phenols in pumpkin seeds was higher than that of free phenolics (Figure 1). The values of total phenols were 2.44–3.82 mg GAE/g pumpkin seed flour, higher than that reported by Marianna who found that of 0.09 to 0.20 mg GAE/g extract. A previous study suggested that the total content of phenolics (TPC) of the seed samples was ranging from 0.95 to 3.43 mg GAE/g DW (20). However, that of *Cucurbita pepo* determined by Peiretti et al. (25) was 9.82 mg GAE/g extract. The differences may be caused by difference in raw materials and extraction methods.

It is obviously that the concentrations of total phenols and flavonoids increased as the rise of temperature (Figure 2). Consequently, the content of total phenolics and flavonoids in pumpkin seeds at 200°C was the highest, which was about 1.56 and 2.81 times of the unroasted one, respectively. Saavedra et al. (20) also found that cooked squash seeds have higher TPC than fresh seeds. Studies have shown that, roasting can increase the total phenolics and flavonoids content of different seeds and grains (19, 26, 27). There are two possible reasons, one is that roasting releases bound phenols and flavonoids because of the destruction of cell structure. The other is that Maillard reaction occurs and produces many compounds, which can react with FC reagent, thus increasing the total phenol content.

Similarly, a higher increase was discovered in antioxidant of pumpkin seeds (Figure 3). The antioxidant properties of cooked pumpkin seeds (0.31–0.32 μmol TE/g) was found to be better than that of fresh seeds (0.37–0.47 μmol TE/g) extracted by methanol (20). Statistical analysis showed that there was a significant correlation between antioxidant activity and total phenol content (*R*^2^ = 0.980, *P* < 0.05). Therefore, the increase of total phenol content is one of the reasons for the enhancement of antioxidant capacity of roasted pumpkin seeds. Otherwise, the generation of Maillard reaction greatly improves the antioxidant properties. Jogihalli et al. (28) considered that is the reason for the increase of antioxidant activity of roasted chickpea. The antioxidant mechanism of Maillard reaction products (MRPs) in food system has been reported and studied; melanin and Amadori rearrangement products (ARP_S_) are considered to have the ability of chelating metals and scavenging oxygen free radicals (29–32). Nooshkam and Madadlou (33) also found that peptides–lactulose system produced more MRPs and presented higher DPPH radical-scavenging activity than whey proteins–lactulose system.

### Phenolic Acids Composition

**Figure 4:**
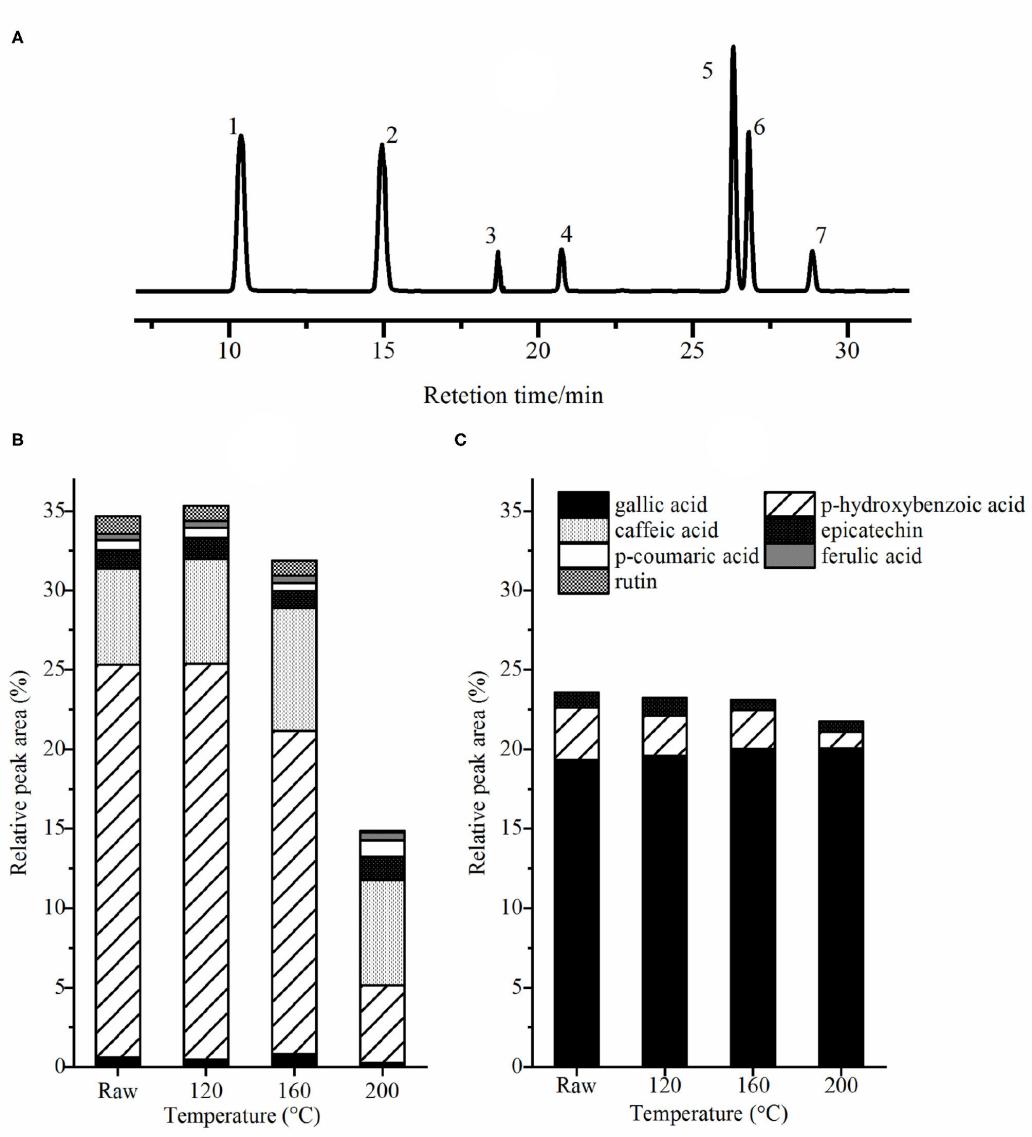
The liquid chromatogram of mixed standard sample **(A)** and the relative percentage of free phenolic acids **(B)** and bound phenolic acids **(C)**. Peaks identification: (1) gallic acid; (2) p-hydroxybenzoic acid; (3) caffeic acid; (4) epicatechin; (5) p-coumarinic acid; (6) ferulic acid; (7) rutint. Raw, raw pumpkin seed.

The liquid chromatogram of mixed standards samples and the relative percentage of different phenolic acids in roasted pumpkin seeds are revealed in Figures 4A–C. The concentrations of p-hydroxybenzoic acids, p-hydroxybenzaldehyde, caffeic acids, and trans-p-coumaric acids was found to be relatively high in cooked pumpkin seeds (20). Gallic acid, p-hydroxybenzoic acid and epicatechin with high total content belong to the derivatives of benzoic acid, while p-coumaric acid, caffeic acid, and ferulic acid are the derivatives of cinnamic acid (8). Caffeic acid and gallic acid are common abundant phenolic acids, which can be esterified with quinine (34). Epicatechin is one of the main catechins in green tea, which has antioxidant, anticancer and metal corrosion inhibition effects (35). Ferulic acid mainly exists in cereals, which is esterified into hemicellulose in cell wall, and has the pharmacological effects of anti platelet aggregation and enhancing the activity of prostaglandins (8). Rutin, a flavonoid extracted from plants, has good antioxidant, antihypertensive and anti-inflammatory effects (36). It was apparent that the content of bound gallic acid was higher than the free one. Conversely, the content of free p-hydroxybenzoic acids was more than the bound ones. In addition, esterified caffeic acids, p-coumaric acids, ferulic acids and rutin in this study were not detected, it may be caused by the difference in species. After roasting, the contents of gallic acid and ferulic acid increased; on the contrary, the contents of p-hydroxybenzoic acid and rutin decreased. In addition, epicatechin and p-coumaric acid were slightly increased, whereas their content was the lowest at 160°C. The decrease of phenolic acid content with baking is due to their thermal instability, while the increase of gallic acid content may be owing to the degradation of polymerized polyphenols (37).

### Fatty Acids Composition

As shown in the Table 1, there are four main fatty acids, namely palmitic acids (C16:0), stearic acids (C18:0), oleic acids (C18:1), and linoleic acids (C18:2) in pumpkin seeds. Among them, oleic acid and linoleic acid are polyunsaturated fatty acids, which have many health benefits such as prevention of cardiovascular disease, promotion of brain and nervous system development (1). Analysis of the relative concentration of fatty acids in pumpkin seeds showed that the unsaturated fatty acids account for more than 83%, and roasting temperature had no evident dependence on the fatty acids of pumpkin seeds (Table 1). The results were close to that of Murkovic et al. (38). Results of Hiromi reflected that there were differences in fatty acid content among different varieties of pumpkin seeds and the content of linoleic acid had no significant loss with a short exposure (12 min) to microwave (39). However, the content of fatty acids in peanut was found to decrease at 200°C roasting for 50 min by Rodrigues et al. (40). As a result, the effect of more roasting time needs to be studied.

**Table 1:** Fatty acid composition of unroasted and roasted pumpkin seeds.

**Roasting temperature (^**°**^C)**	**Peak area ratio (%)**					
	**C16:0**	**C18:0**	**C18:1**	**C18:2**	**Saturated fatty acids**	**Unsaturated fatty acids**
Raw pumpkin seed	11.55 ± 0.13	5.21 ± 0.19	25.21 ± 0.33	56.90 ± 0.64	16.76 ± 0.31	82.11 ± 0.30
120	11.43 ± 0.04	4.94 ± 0.07	24.63 ± 0.19	57.86 ± 0.30	16.37 ± 0.11	82.50 ± 0.11
160	11.51 ± 0.05	5.16 ± 0.05	25.29 ± 0.13	56.88 ± 0.20	16.67 ± 0.09	82.50 ± 0.11
200	11.53 ± 0.03	4.88 ± 0.02	24.55 ± 0.04	57.91 ± 0.03	16.41 ± 0.01	82.46 ± 0.01

### Volatile Matter

**Figure 5:**
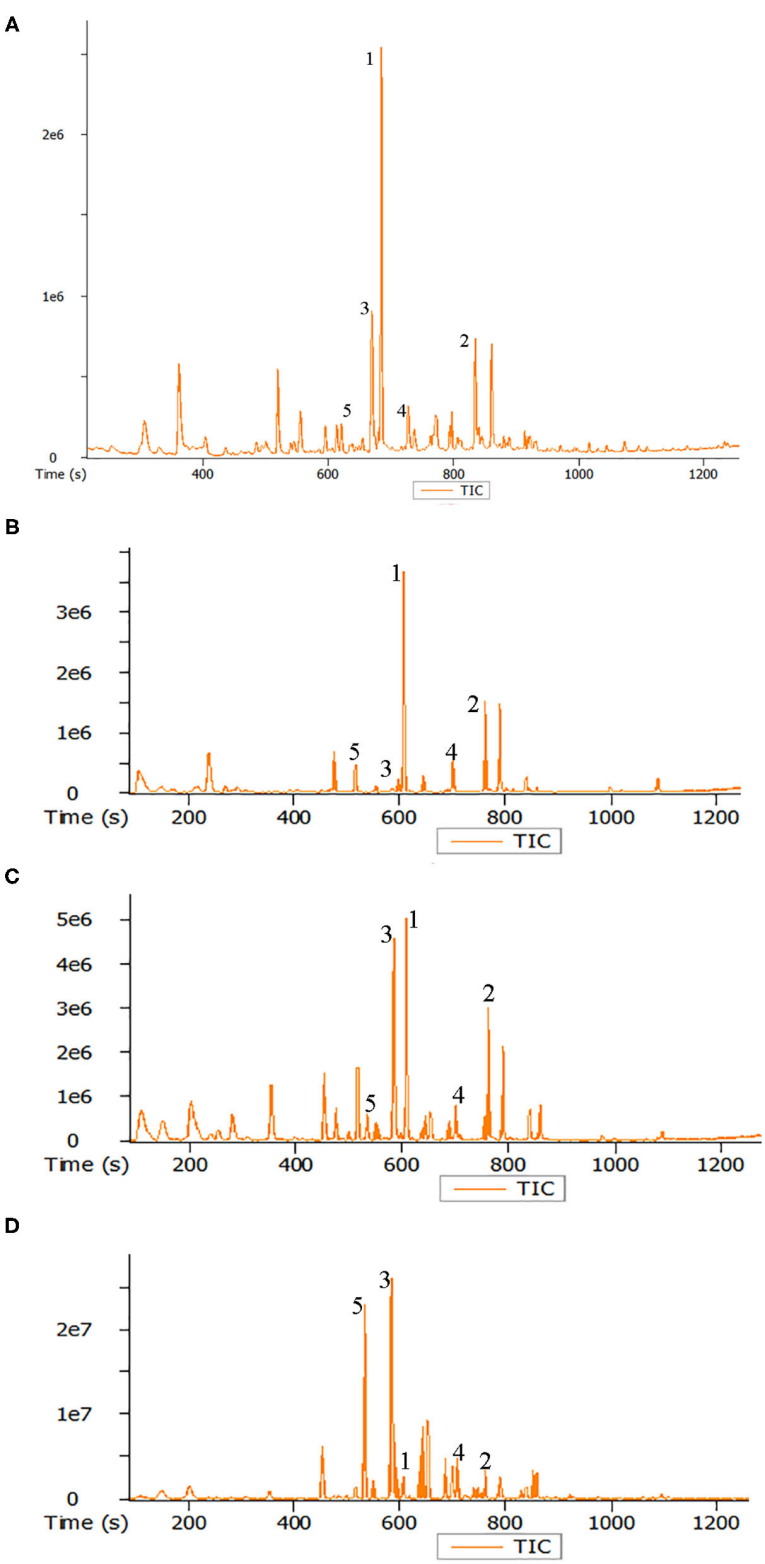
Total ion flow diagram of volatile components in raw pumpkin seeds **(A)** and roasted pumpkin seeds at 120°C **(B)**, 160°C **(C)**, and 200°C **(D)**. Peaks identification: (1) n-hexanol; (2) 2,3-butanediol; (3) 2,5-dimethylpyrazine; (4) acetic acid; (5) 2-methylpyrazine.

A number of volatile compounds could be identified in roasted seeds, including heterocyclic compounds, aldehydes, ketones, alcohols, acids, esters, and sulfur compounds. Twenty-nine volatile matters with higher relative concentration in roasted pumpkin seeds were listed in Table 2 and the total ion flow diagram of volatile components in raw and roasted pumpkin seeds are shown in Figures 5A–D.

**Table 2:** Volatile compounds of unroasted and roasted pumpkin seeds.

**Name**	**Peak area ratio (%)**			
	**Raw pumpkin seed**	**120^**°**^C**	**160^**°**^C**	**200^**°**^C**
**Heterocycles**				
Pyrazine	–	0.11 ± 0.01^b^	0.41 ± 0.05^b^	0.58 ± 0.52^a^
2-Methylpyrazine	–	0.38 ± 0.33^c^	1.27 ± 0.30^b^	17.68 ± 0.14^a^
2-Ethylpyrazine	–	–	–	0.93 ± 0.08
2,5-Dimethylpyrazine	4.23 ± 3.80^c^	–	8.39 ± 0.38^b^	17.13 ± 0.61^a^
2,6-Dimethylpyrazine	–	–	0.15 ± 0.13^b^	3.25 ± 0.05^a^
2,3-Dimethylpyrazine	0.18 ± 0.03^b^	–	0.09 ± 0.00^c^	0.79 ± 0.02^a^
2-Ethyl-5-methylpyrazine	–	–	0.90 ± 0.04^b^	3.48 ± 0.25^a^
3-Ethyl-2,5-dimethylpyrazine	–	–	0.28 ± 0.01^b^	1.48 ± 0.09^a^
2,3,5-Trimethylpyrazine	–	–	1.06 ± 0.06^b^	3.85 ± 0.20^a^
Pyridine	–	0.03 ± 0.02^c^	0.15 ± 0.01^b^	1.35 ± 0.09^a^
2-Acetylpyrrole	–	0.01 ± 0.00^c^	0.03 ± 0.01^b^	0.13 ± 0.02^a^
2-Pentylfuran	–	0.57 ± 0.29^a^	0.59 ± 0.02^a^	0.39 ± 0.28^a^
2-Acetylfuran	–	–	0.03 ± 0.00^b^	0.80 ± 0.04^a^
5-Methyl furfural	–	–	–	0.35 ± 0.41
Furfural	–	0.01 ± 0.00^b^	0.17 ± 0.16^b^	1.96 ± 0.33^a^
2-Methylthiazole	–	0.01 ± 0.00^b^	0.01 ± 0.00^b^	0.17 ± 0.01^a^
**Alcohols**				
2,3-Butanediol	7.98 ± 00.59^b^	11.13 ± 1.36^a^	8.90 ± 0.40^b^	2.33 ± 0.13^c^
Methyl mercaptan	–	0.53 ± 0.20^a^	0.33 ± 0.67^a^	0.27 ± 0.14^b^
n-Hexanol	–	14.72 ± 1.74^a^	7.12 ± 0.68^b^	0.85 ± 0.07^c^
3-Methyl-1-butanol	–	2.16 ± 0.62^a^	1.00 ± 0.03^b^	0.17 ± 0.01^c^
Benzyl alcohol	0.34 ± 0.26^a^	0.22 ± 0.33^b^	0.07 ± 0.00^c^	–
**Acids**				
2-Methylbutyric acid	0.34 ± 0.01^c^	0.41 ± 0.04^b^	1.02 ± 0.04^a^	–
Acetic acid	1.86 ± 0.39^a^	2.40 ± 0.90^a^	1.80 ± 0.18^a^	1.11 ± 0.96^a^
**Ketones**				
2-Heptanone	–	0.04 ± 0.03^c^	1.27 ± 0.03^b^	17.68 ± 0.15^a^
Acetylacetone	–	–	0.31 ± 0.02^b^	1.24 ± 0.06^a^
1-Hydroxy-2-propanone	–	0.07 ± 0.04^c^	0.55 ± 0.08^a^	0.26 ± 0.02^b^
2,3-Pentanedione	–	–	0.24 ± 0.01^a^	0.18 ± 0.02^b^
**Aldehyde**				
hexanal	–	0.13 ± 0.07^c^	3.30 ± 0.28^a^	0.69 ± 0.07^b^
benzaldehyde	0.39 ± 0.07^b^	0.17 ± 0.03^c^	0.68 ± 0.04^a^	0.20 ± 0.02^c^

*Values with similar superscripts in a column do not differ significantly (P < 0.05). –, not detected*.

The following pyrazines were identified in the roasted pumpkin seeds: pyrazine, 2-methylpyrazine, 2-ethylpyrazine, 2,5-dimethylpyrazine, 2,6-dimethylpyrazine, 2,3-dimethylpyrazine, 2-ethyl-5-methylpyrazine, 3-ethyl-2,5-dimethylpyrazine, and 2,3,5-trimethylpyrazine. It can be seen from the table that the concentration of pyrazines in the pumpkin seeds increase significantly with the increase of temperature. They have been reported as typical Maillard reaction products and the pyrazines were considered to be the important factor of the characteristic aroma of pumpkin seeds oil (5, 41, 42). In addition, 2-acetylpyrrole originating from the Maillard reaction is also known for intensifying the flavor of pumpkin seed oil (5, 42). The four furan derivatives detected were 2-pentylfuran, 2-acetylfuran, 5-methylfurfural and furfural. None of the four furan derivatives were detected in pumpkin seeds and they showed an increasing trend with the high of temperature. Lipid peroxide or carbohydrate degradation were two possible reasons for the formation of furan derivatives (11). They have also been found in other roasted seeds and may be the important component the characteristic roasted, nut burnt aroma (43, 44).

There were two main aldehydes in the headspace of the pumpkin seeds. Benzaldehyde is considered to be the degradation product of amino acid phenylalanine (41); hexanal comes from linoleic oxidation (42). Interestingly, the content of these two aldehydes was the highest at 160°C. In addition, five alcohols were found. With the increase of roasting temperature, the content of these alcohols decreased significantly. The short-chain linear alcohols were derived from lipid oxidation and 3-methyl-1-butanol was probably the product of Strecker degradation (45). In general, the changes of these volatile compounds were mainly due to Maillard reaction and made roasted pumpkin seeds present toast tan and rich caramel aroma.

### Protein Electrophoresis

**Figure 6:**
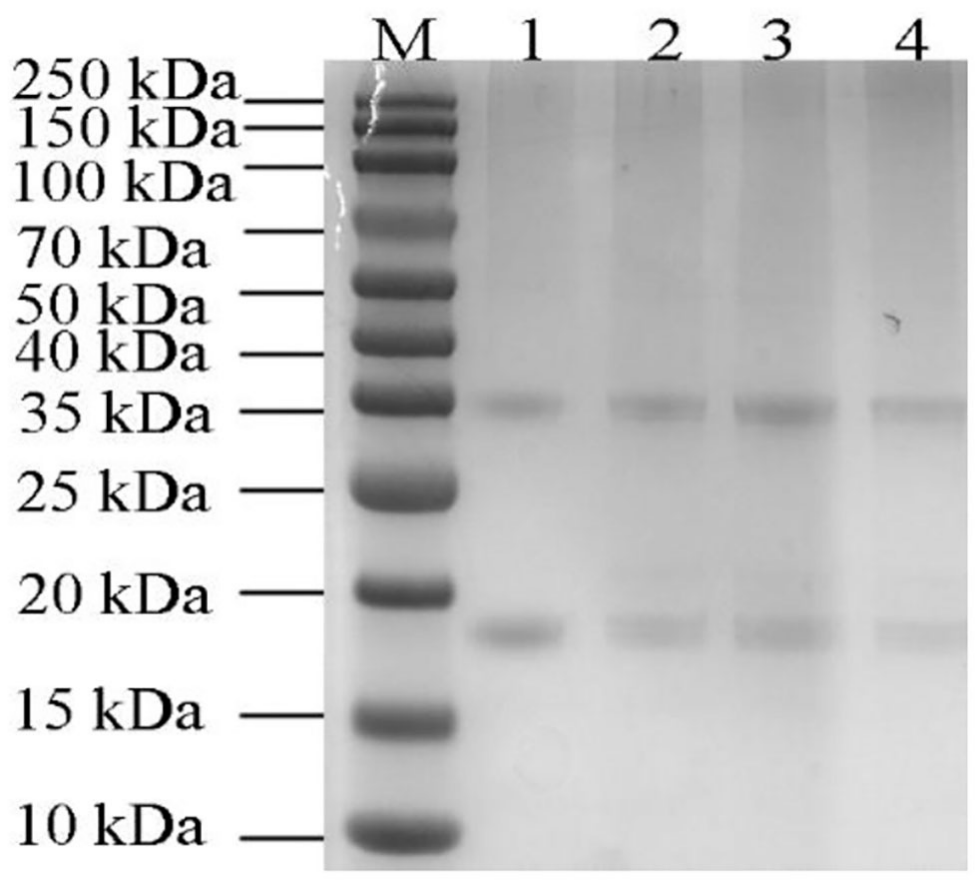
SDS-PAGE of total pumpkin seeds protein (5 μg) at different roasted temperatures. Lane M, marker; Lane 1, unroasted pumpkin seeds protein; Lane 2–4, roasted pumpkin seeds protein at 120, 160, and 200°C.

As shown in Figure 6, the major molecular weight of pumpkin seeds protein isolate was 35 and 20 kDa, which was consistent with that of Yang et al. (46). Only few literatures reported that the characteristic fraction of pumpkin seed protein isolate is 12S globulin and the subunit of globulin is composed of two polypeptides of about 30–40 and 20 kDa by disulfide bond (17, 24). Other less content fractions are albumin, prolamin, and glutelin (24, 47). No remarkable changes of the protein bands have been observed from those roasted with lower temperature; while the intensity of which decreased when the roasting temperature reached 200°C. High temperature leads to higher degree of protein denaturation, resulting in the decrease of protein band intensity at 200°C. Wang et al. (48) found lighter soy protein bands with increasing preheating temperature and time because of higher denaturation. Mohamed Ahmed et al. (49) considered that low temperature and short roasting time had no effect on Samh seeds protein bands according to the results of his experiment. Our result may indicate that heating of pumpkin seed to the temperature as high as 200°C did not significantly affect the molecular weight distribution of pumpkin seed protein hydrolysate. Furthermore, the similarity of globulin structure between pumpkin seed protein and bean protein and the low allergenicity of pumpkin seeds make it possible to replace soybean protein in food formulation (1). Pumpkin seed proteins and peptides have antioxidant, antifungal, antihypertensive, and other biological activities (50–52). Therefore, they can be used as substrates for the research of biological complexes in food and pharmaceutical products, as well as excellent alternative sources of proteins in the bread and meat industries (47).

### Changes of Particle Size

After 4 h, the particle size of each pumpkin seed solution increased (Table 3), which suggested that pumpkin seeds protein was unstable. With the increase of time, the aggregates grew and precipitated. The particle size of protein solution roasted at 120 and 200°C were higher than that of unroasted protein. On the contrary, the protein roasted at 160°C had the similar particle size with the raw one, so it showed relatively high stability. The result indicated that the suitable roasting temperature was conducive to obtain heat stable pumpkin seed protein isolate. On the other hand, protein particles with adjustable size and aggregation rate can be obtained by preheating treatment, which can be combined with emulsification technology to prepare heat stable protein (48, 53). Wang et al. (48) reported that the stable soy protein isolate (SPI) could be obtained with high preheating temperature and enough preheating time, with provided a new perspective for preparing heat stable SPI for better utilization. The result of this study indicates that pumpkin seed protein may be used as a potential stabilizer in the food products whose processing may require high temperature.

**Table 3:** Change of particle size of pumpkin seeds protein in pure water.

**Temperature (^**°**^C)**	**Mean diameter (nm)**	
	**0 h**	**4 h**
Raw pumpkin seed	532.53 ± 41.68^c^	628.67 ± 55.37^b^
120	831.49 ± 74.80^a^	904.16 ± 40.92^a^
160	488.50 ± 36.07^c^	508.26 ± 31.45^c^
200	731.85 ± 35.62^b^	967.39 ± 13.47^a^

*Different small letters (a–d) indicate that there are significant differences in the particle size at different roasting temperatures (P < 0.05)*.

## Conclusion

In conclusion, roasting at certain temperature can increase the sensory and nutritional attributes of pumpkin seed. After roasting, the concentrations of total phenols, total flavonoids, and antioxidant properties of pumpkin seeds were improved, which indicated that roasting enhanced the biological activity and antioxidant properties of pumpkin seeds. A large number of pyrazine compounds were produced in roasted pumpkin seeds, contributing to strong roasted food aroma. The main fatty acids components of pumpkin seeds were C16:0, C18:0, C18:1, and C18:2 and roasting temperature has no apparent influence on concentrations of saturated and unsaturated fatty acids. Although the properties of pumpkin seed protein increased after roasting, the result of protein electrophoresis and particle size showed that 160°C was better than 200°C. In addition, the effect of roasting on the structure and functionality of pumpkin seed protein needs to be further confirmed. A detailed understanding between structure change after roasting and functionality of protein will enable researchers to develop pumpkin seed protein-based food products for different purposes.

## Data Availability Statement

The raw data supporting the conclusions of this article will be made available by the authors, without undue reservation.

## Author Contributions

MP: conceptualization, investigation, methodology, software, data curation, writing – original draft, and writing – review and editing. DL: investigation. BJ: formal analysis, investigation, and writing – review and editing. JC: project administration, funding acquisition, and writing – review and editing. All authors: contributed to the article and approved the submitted version.

## Conflict of Interest

The authors declare that the research was conducted in the absence of any commercial or financial relationships that could be construed as a potential conflict of interest.

## References

[1] Dotto JM Chacha JS. The potential of pumpkin seeds as a functional food ingredient: a review. Sci. Afr. (2020) 10:e00575. 10.1016/j.sciaf.2020.e00575

[2] Xanthopoulou MN Nomikos T Fragopoulou E Antonopoulou S. Antioxidant and lipoxygenase inhibitory activities of pumpkin seed extracts. Food Res. Int. (2009) 42:641–6. 10.1016/j.foodres.2009.02.003

[3] Wang L Liu F Wang A Yu Z Xu Y Yang Y. Purification, characterization and bioactivity determination of a novel polysaccharide from pumpkin (Cucurbita *moschata*) seeds. Food Hydrocolloids. (2017) 66:357–64. 10.1016/j.foodhyd.2016.12.003

[4] Nederal S Petrović M Vincek D Pukec D Škevin D Kraljić K. Variance of quality parameters and fatty acid composition in pumpkin seed oil during three crop seasons. Ind. Crops Prod. (2014) 60:15–21. 10.1016/j.indcrop.2014.05.044

[5] Potočnik T Rak Cizej M Košir IJ. Influence of seed roasting on pumpkin seed oil tocopherols, phenolics and antiradical activity. J. Food Compos. Anal. (2018) 69:7–12. 10.1016/j.jfca.2018.01.020

[6] Abdel Aziz AR AbouLaila MR Aziz M Omar MA Sultan K. *In vitro* and *in vivo* anthelmintic activity of pumpkin seeds and pomegranate peels extracts against *Ascaridia galli*. Beni-Suef Univ. J. Basic Appl. Sci. (2018) 7:231–4. 10.1016/j.bjbas.2018.02.003

[7] Jacobo-Valenzuela N Maróstica-Junior MR Zazueta-Morales JdJ Gallegos-Infante JA. Physicochemical, technological properties, and health-benefits of *Cucurbita moschata* Duchense vs Cehualca: a review. Food Res. Int. (2011) 44:2587–93. 10.1016/j.foodres.2011.04.039

[8] Dai J Mumper R. Plant phenolics: extraction, analysis and their antioxidant and anticancer properties. Molecules (Basel, Switzerland). (2010) 15:7313–52. 10.3390/molecules15107313 20966876 PMC6259146

[9] Peričin D Krimer V Trivić S Radulović L. The distribution of phenolic acids in pumpkin's hull-less seed, skin, oil cake meal, dehulled kernel and hull. Food Chem. (2009) 113:450–6. 10.1016/j.foodchem.2008.07.079

[10] Enneb S Drine S Bagues M Triki T Boussora F Guasmi F. Phytochemical profiles and nutritional composition of squash (*Cucurbita moschata* D.) from Tunisia. S Afr J Bot. (2020) 130:165–71. 10.1016/j.sajb.2019.12.011

[11] Siegmund B Murkovic M. Changes in chemical composition of pumpkin seeds during the roasting process for production of pumpkin seed oil (Part 2: volatile compounds). Food Chem. (2004) 84:367–74. 10.1016/S0308-8146(03)00241-3

[12] Poehlmann S Schieberle P. Characterization of the aroma signature of styrian pumpkin seed oil (*Cucurbita pepo* subsp. pepo var Styriaca*)* by Molecular Sensory Science. J Agric Food Chem. (2013) 61:2933–42. 10.1021/jf400314j 23461409

[13] Potočnik T Kosir I. Influence of roasting temperature of pumpkin seed on PAH and aroma formation: influence of roasting temperature on PAH and aroma. Eur J Lipid Sci Technol. (2016) 119:1500593. 10.1002/ejlt.201500593

[14] Sá AGA Moreno YMF Carciofi BAM. Plant proteins as high-quality nutritional source for human diet. Trends Food Sci Technol. (2020) 97:170–84. 10.1016/j.tifs.2020.01.011

[15] Vinayashree S Vasu P. Biochemical, nutritional and functional properties of protein isolate and fractions from pumpkin (*Cucurbita moschata* var. Kashi Harit) seeds. Food Chem. (2021) 340:128177. 10.1016/j.foodchem.2020.128177 33002826

[16] Glew RH Glew RS Chuang LT Huang YS Millson M Constans D. Amino acid, mineral and fatty acid content of pumpkin seeds (*Cucurbita* spp) and *Cyperus esculentus* nuts in the Republic of Niger. Plant Foods Hum Nutr. (2006) 61:49–54. 10.1007/s11130-006-0010-z 16770692

[17] Rezig L Chibani F Chouaibi M Dalgalarrondo M Hessini K Guéguen J. Pumpkin (*Cucurbita maxima*) seed proteins: sequential extraction processing and fraction characterization. J Agric Food Chem. (2013) 61:7715–21. 10.1021/jf402323u 23869935

[18] Dash P Ghosh G. Amino acid composition, antioxidant and functional properties of protein hydrolysates from Cucurbitaceae seeds. J Food Sci Technol. (2017) 54:4162–72. 10.1007/s13197-017-2855-6 29184221 PMC5685994

[19] Bhinder S Singh B Kaur A Singh N Kaur M Kumari S. Effect of infrared roasting on antioxidant activity, phenolic composition and Maillard reaction products of Tartary buckwheat varieties. Food Chem. (2019) 285:240–51. 10.1016/j.foodchem.2019.01.141 30797341

[20] Saavedra MJ Aires A Dias C Almeida JA De Vasconcelos MCBM Santos P. Evaluation of the potential of squash pumpkin by-products (seeds and shell) as sources of antioxidant and bioactive compounds. J Food Sci Technol. (2015) 52:1008–15. 10.1007/s13197-013-1089-5 25694712 PMC4325009

[21] Shen Y Jin L Xiao P Lu Y Bao J. Total phenolics, flavonoids, antioxidant capacity in rice grain and their relations to grain color, size and weight. J Cereal Sci. (2009) 49:106–11. 10.1016/j.jcs.2008.07.010

[22] Sánchez-Moreno C Larrauri JA Saura-Calixto F. A procedure to measure the antiradical efficiency of polyphenols. J Sci Food Agric. (1998) 76:270–6. 10.1002/(SICI)1097-0010(199802)76:2<270::AID-JSFA945>3.0.CO;2-9

[23] Zhang S Zu Y-G Fu Y-J Luo M Liu W Li J. Supercritical carbon dioxide extraction of seed oil from yellow horn (*Xanthoceras sorbifolia* Bunge.) and its anti-oxidant activity. Bioresour Technol. (2009) 101:2537–44. 10.1016/j.biortech.2009.11.082 20022744

[24] Bučko S Katona J Popović L Vaštag Ž Petrovic L Vucinic Vasic M. Investigation on solubility, interfacial and emulsifying properties of pumpkin (*Cucurbita pepo*) seed protein isolate. LWT Food Sci Technol. (2015) 64:609–15. 10.1016/j.lwt.2015.06.054

[25] Peiretti PG Meineri G Gai F Longato E Amarowicz R. Antioxidative activities and phenolic compounds of pumpkin (*Cucurbita pepo*) seeds and amaranth (*Amaranthus caudatus*) grain extracts. Nat Prod Res. (2017) 31:2178–82. 10.1080/14786419.2017.1278597 28114838

[26] Gallegos-Infante J Rocha-Guzmán N González-Laredo R Pulido-Alonso J. Effect of processing on the antioxidant properties of extracts from Mexican barley (*Hordeum vulgare*) cultivar. Food Chem. (2010) 119:903–6. 10.1016/j.foodchem.2009.07.044

[27] Ee KY Agboola S Rehman A Zhao J. Characterisation of phenolic components present in raw and roasted wattle (*Acacia victoriae* Bentham) seeds. Food Chem. (2011) 129:816–21. 10.1016/j.foodchem.2011.05.028 25212304

[28] Jogihalli P Singh L Sharanagat VS. Effect of microwave roasting parameters on functional and antioxidant properties of chickpea (*Cicer arietinum*). LWT Food Sci Technol. (2017) 79:223–33. 10.1016/j.lwt.2017.01.047 28763959

[29] Woffenden HM Ames JM Chandra S Anese M Nicoli MC. Effect of kilning on the antioxidant and pro-oxidant activities of pale malts. J Agric Food Chem. (2002) 50:4925–33. 10.1021/jf020312g 12166984

[30] Lertittikul W Benjakul S Tanaka M. Characteristics and antioxidative activity of Maillard reaction products from a porcine plasma protein-glucose model system as influenced by pH. Food Chem. (2007) 100:669–77. 10.1016/j.foodchem.2005.09.085

[31] Michalska A Amigo-Benavent M Zielinski H del Castillo MD. Effect of bread making on formation of Maillard reaction products contributing to the overall antioxidant activity of rye bread. J Cereal Sci. (2008) 48:123–32. 10.1016/j.jcs.2007.08.012

[32] Nooshkam M Varidi M Bashash M. The Maillard reaction products as food-born antioxidant and antibrowning agents in model and real food systems. Food Chem. (2019) 275:644–60. 10.1016/j.foodchem.2018.09.083 30724245

[33] Nooshkam M Madadlou A. Maillard conjugation of lactulose with potentially bioactive peptides. Food Chem. (2016) 192:831–6. 10.1016/j.foodchem.2015.07.094 26304417

[34] Pietta P Minoggio M Bramati L. Plant polyphenols: structure, occurrence and bioactivity. In: ed. Rahman A-u editor. Studies in Natural Products Chemistry. Amsterdam: Elsevier (2003). p. 257–312.

[35] Espinoza Vázquez A Figueroa IA Gómez FJR Vásquez AP Mata R Ángeles Beltrán D. (-) - Epicatechin gallate as a corrosion inhibitor for bronze in a saline medium and theoretical study. J Mol Struct. (2020) 1227:129416. 10.1016/j.molstruc.2020.129416

[36] Kalinová JP Vrchotová N Tríska J. Contribution to the study of rutin stability in the achenes of Tartary buckwheat (*Fagopyrum tataricum*). Food Chem. (2018) 258:314–20. 10.1016/j.foodchem.2018.03.090 29655739

[37] Alamprese C Ratti S Rossi M. Effects of roasting conditions on hazelnut characteristics in a two-step process. J Food Eng. (2009) 95:272–9. 10.1016/j.jfoodeng.2009.05.001

[38] Murkovic M Piironen V Lampi AM Kraushofer T Sontag G. Changes in chemical composition of pumpkin seeds during the roasting process for production of pumpkin seed oil (Part 1: non-volatile compounds). Food Chem. (2004) 84:359–65. 10.1016/S0308-8146(03)00240-1

[39] Yoshida H Tomiyama Y Hirakawa Y Mizushina Y. Microwave roasting effects on the oxidative stability of oils and molecular species of triacylglycerols in the kernels of pumpkin (*Cucurbita* spp.) seeds. J Food Compos Anal. (2006) 19:330–9. 10.1016/j.jfca.2004.10.004

[40] Rodrigues AC Ströher GL Freitas AR Visentainer JV Oliveira CC de Souza NE. The effect of genotype and roasting on the fatty acid composition of peanuts. Food Res Int. (2011) 44:187–92. 10.1016/j.foodres.2010.10.042

[41] Ames JM Guy RCE Kipping GJ. Effect of pH, temperature, and moisture on the formation of volatile compounds in glycine/glucose model systems. J Agric Food Chem. (2001) 49:4315–23. 10.1021/jf010198m 11559131

[42] Liu X Jin Q Liu Y Huang J Wang X Mao W. Changes in volatile compounds of peanut oil during the roasting process for production of aromatic roasted peanut oil. J Food Sci. (2011) 76:C404–12. 10.1111/j.1750-3841.2011.02073.x 21535807

[43] Jung MY Bock JY Back SO Lee TK Kim JH. Pyrazine contents and oxidative stabilities of roasted soybean oils. Food Chem. (1997) 60:95–102. 10.1016/S0308-8146(96)00316-0

[44] Kiralan M. Volatile compounds of black cumin seeds (*Nigella sativa* L.) from microwave-heating and conventional roasting. J Food Sci. (2012) 77:C481–4. 10.1111/j.1750-3841.2012.02638.x 22515239

[45] Wu S Xu T Akoh CC. Effect of roasting on the volatile constituents of *Trichosanthes kirilowii* seeds. J Food Drug Anal. (2014) 22:310–7. 10.1016/j.jfda.2013.12.005 28911420 PMC9354876

[46] Yang C Wang B Wang J Xia S Wu Y. Effect of pyrogallic acid (1,2,3-benzenetriol) polyphenol-protein covalent conjugation reaction degree on structure and antioxidant properties of pumpkin (*Cucurbita* sp.) seed protein isolate. LWT Food Sci. Technol. (2019) 109:443–9. 10.1016/j.lwt.2019.04.034

[47] Kotecka-Majchrzak K Sumara A Fornal E Montowska M. Oilseed proteins - properties and application as a food ingredient. Trends Food Sci Technol. (2020) 106:160–70. 10.1016/j.tifs.2020.10.004

[48] Wang J Burton Navicha W Na X Ma W Xu X Wu C. Preheat-induced soy protein particles with tunable heat stability. Food Chem. (2021) 336:127624. 10.1016/j.foodchem.2020.127624 32768901

[49] Mohamed Ahmed IA Al Juhaimi FY Osman MA Al Maiman SA Hassan AB Alqah HAS. Effect of oven roasting treatment on the antioxidant activity, phenolic compounds, fatty acids, minerals, and protein profile of Samh (*Mesembryanthemum forsskalei* Hochst) seeds. LWT Food Sci Technol. (2020) 131:109825. 10.1016/j.lwt.2020.109825

[50] Vassiliou AG Neumann GM Condron R Polya GM. Purification and mass spectrometry-assisted sequencing of basic antifungal proteins from seeds of pumpkin (*Cucurbita maxima*). Plant Sci. (1998) 134:141–62. 10.1016/S0168-9452(98)00052-1

[51] Nkosi CZ Opoku AR Terblanche SE. Effect of pumpkin seed (*Cucurbita pepo*) protein isolate on the activity levels of certain plasma enzymes in CCl4-induced liver injury in low-protein fed rats. Phytother Res. (2005) 19:341–5. 10.1002/ptr.1685 16041732

[52] Nkosi CZ Opoku AR Terblanche SE. *In vitro* antioxidative activity of pumpkin seed (Cucurbita pepo) protein isolate and its *in vivo* effect on alanine transaminase and aspartate transaminase in acetaminophen-induced liver injury in low protein fed rats. Phytother Res. (2006) 20:780–3. 10.1002/ptr.1958 16807884

[53] Nicolai T Durand D. Controlled food protein aggregation for new functionality. Curr Opin Colloid Interf Sci. (2013) 18:249–56. 10.1016/j.cocis.2013.03.001

